# Anti-CD74 IgA is not associated with modic changes in the Northern Finland Birth Cohort

**DOI:** 10.1007/s10067-026-07985-4

**Published:** 2026-02-11

**Authors:** Annik Steimer, Friederike Schulze, Juhani Määttä, Mika Nevalainen, Jaro Karppinen, Stefan Dudli, Adrian Ciurea

**Affiliations:** 1 Center of Experimental Rheumatology, Department of Rheumatology, University Hospital Zurich, University of Zurich, Zurich, Switzerland; 2 Department of Physical Medicine and Rheumatology, Balgrist University Hospital, Balgrist Campus, University of Zurich, Zurich, Switzerland; 3 Research Unit of Health Sciences and Technology, University of Oulu, Oulu, Finland; 4 Medical Research Center Oulu, University of Oulu and Oulu University Hospital, Oulu, Finland; 5 Wellbeing Services County of South Karelia, Lappeenranta, Finland

**Keywords:** Axial spondyloarthritis, Biomarker, IgA anti-CD74 antibodies, MC

## Abstract

**Objectives:**

Chronic low back pain is a common symptom in axial spondyloarthritis (axSpA) and in patients with degenerative spinal disease associated with Modic changes (MC) on MRI. A clear distinction between axSpA and MC might be difficult in early disease, particularly in the absence of axSpA sacroiliac changes. Therefore, a diagnostic serum biomarker for ambiguous cases would be useful. To evaluate if the potential diagnostic biomarker anti-CD74 for axSpA is elevated in patients with MC as well, we measured this antibody in patients with MC and healthy controls. We presumed that a negative anti-CD74 antibody in MC would be helpful to distinguish it from axSpA.

**Method:**

Participants from the NFBC1966 with MC and matched participants without MC were included in this study. Anti-CD74 immunoglobulin A (IgA) was measured with a commercially available kit. An association between continuous log-transformed anti-CD74 IgA was evaluated with a linear regression model adjusted for body mass index (BMI), sex, C-reactive protein (CRP), and presence of low back pain.

**Results:**

The study involved 307 participants, 148 with MC and 159 without MC. There was no association between MC and continuous anti-CD74 IgA after adjustment for potential confounders (Exp(B) = 1.3, 95% CI 0.8 to 2.1). None of the participants with MC had elevated anti-CD74 IgA above the cut-off of 15 U/ml.

**Conclusions:**

Anti-CD74 IgA is not elevated in patients with MC. Further studies comparing directly patients with axSpA and MC are required to evaluate if it is a diagnostic tool to differentiate axSpA and MC in unclear cases.

**Table:** 

**Key Points** • *Anti-CD74 IgA are not elevated in patients with Modic changes on MRI.*

**Supplementary Information:**

The online version contains supplementary material available at https://doi.org/10.1007/s10067-026-07985-4.

## Introduction

Axial spondyloarthritis (axSpA) is a chronic inflammatory rheumatic disease that primarily affects the axial skeleton (the sacroiliac joints (SIJs) and the spine) in young individuals [1]. While magnetic resonance imaging (MRI) has allowed a timely diagnosis, it has also been a possible source of misdiagnosis and treatment failure [2, 3]. The presence of bone marrow edema at the level of the SIJs might be unspecific and several axSpA mimics, for example, degenerative changes called Modic changes (MC), have been described [4]. A minority of axSpA patients (< 10%) have an inflammatory involvement of the spine without SIJ affection. The differential diagnosis in these patients with anterior or posterior vertebral corner edema on MRI includes the presence of incipient degenerative disease. The initial stages of MC, vertebral alterations adjacent to degenerated intervertebral discs observed on MRI, may be located at the vertebral corner and may not be necessarily associated with obvious disc alterations on MRI [5]. In the past few years, antibodies against cluster of differentiation 74 (anti-CD74) have been presented as a potential diagnostic serum biomarker for axSpA [6–12], whereas no serum biomarker is available for patients with MC [13]. Previous studies focused on the comparison of anti-CD74 antibodies of patients with axSpA with healthy controls or patients with fibromyalgia [6–12]. One study included patients with degenerative changes in its control group [7], and another one included patients with chronic low back pain [14], none specifically focusing on patients with MC. The availability of a biomarker that allows differentiation between the two disorders might be helpful in specific situations.

In this study, we compare serum levels of participants with MC with matched healthy controls from the Northern Finland Birth Cohort 1966 (NFBC1966) [15, 16]. We hypothesize that there is no anti-CD74 antibody elevation in participants with MC compared to healthy controls, and that anti-CD74 antibody is, in contrast to axSpA, not a serum biomarker for MC.

## Materials and methods

### Study population

This study is a follow-up on a subpopulation of the NFBC1966 [13, 15–17] who underwent MRI and were diagnosed with MC, as well as a matched control group from the same cohort who also received MRI. All participants provided informed consent for data and biomaterial usage. The study was approved by the regional medical research ethics committee of the Wellbeing Services County of North Ostrobothnia, Finland, as well as the ethics commission of the canton of Zurich, Switzerland. Each participant provided a serum sample, underwent lumbar MRI, and completed a set of questionnaires. Data and serum collection were done as specified in the NFBC1966 study protocol, which is available on the cohort’s website. The serum was stored for 11–13 years at –80 °C and went through two shipments on dry ice and three freeze–thaw cycles before the current use in this study [13, 15–17]. The MRI scans with the MC classification and extent of the lesions have previously been reported [13, 17]. All participants filled out questionnaires on comorbidities including rheumatological diseases. In participants with either self-reported axSpA or elevated anti-CD74 antibodies, the MRI was additionally evaluated for specific axSpA features by an experienced radiologist (MN).

### Anti-CD74 IgA measurement

We measured serum anti-CD74 immunoglobulin A (IgA), which has a high specificity in axSpA [6] and a superior diagnostic capability in differentiating patients with axSpA from those with chronic low back pain than, e.g., anti-CD74 IgG [14]. We measured serum anti-CD74 IgA with a commercially available ELISA-based kit (Aesku.diagnostics, Wendelsheim, Germany) according to the manufacturer’s instruction with one exception: we diluted the serum 1:251 instead of 1:1001, to avoid measurements under the detection limit of the kit. It was previously described that anti-CD74 IgA levels above 15 U/ml are suggestive for axSpA [9, 18]. We confirmed the measurements of the four samples with results higher than 15 U/ml and of the four participants with self-reported axSpA with a second measurement. In those samples, we used the mean value for the analysis.

### Statistical analysis

All tests were performed with R version 4.4.1. [19] We report continuous values as mean with standard deviation or as median with interquartile range, respectively. Categorical data are reported as number and percentages. We used multiple linear regression models adjusted for potential confounders (sex, body mass index (BMI), C-reactive protein (CRP), presence of low back pain; the factors known to be potentially associated with the presence of MC and/or elevated anti-CD74 IgA [18, 20]) to evaluate an association between continuous anti-CD74 IgA and MC in MRI. To avoid confounding through self-reported axSpA, we excluded these patients from the association analysis. Additionally, we evaluated the association of anti-CD74 IgA with the different MC types and MC load (a measure for the extent of MCs). CRP and anti-CD74 IgA values were log-transformed (after addition of 0.001) for the regression analysis. A *p*-value below 0.05 was considered statistically significant.

## Results

Three hundred seven participants from the NFBC1966 had available blood samples. Forty-eight percent of these participants had MC on MRI with mixed type 1 and type 2 MC in 91%. Low back pain was reported by 29% of all included participants. Some participants self-reported rheumatic diseases (Table 1).

**Table 1:** Baseline characteristics and self-reported rheumatic comorbidities

	MC (*n* = 148)	Without MC (*n* = 159)	*p*-value
MC type, *N* (%) MC1 MC2 MC3 MC1/MC2 MC1/MC2/MC3	12 (8.1) 1 (0.7) 0 (0.0) 134 (90.5) 1 (0.7)		** < 0.001**
Male, *N* (%)	65 (43.9)	49 (30.8)	**0.02**
BMI, kg/m^2^ (median [IQR])	25.4 [23.4, 27.7]	25.4 [23.3, 28.4]	0.91
Anti-CD74 IgA, U/ml (median [IQR])	1.5 [0.7, 3.0]	1.6 [0.6, 2.9]	0.62
Anti-CD74 IgA elevated, *N* (%)	0 (0.0)	4 (2.5)	0.12
CRP, mg/l (median [IQR])	1.2 [0.5. 2.4]	1.0 [0.5, 3.0]	0.77
Low back pain, *N* (%)	42 (29.0)	47 (30.1)	0.90
AxSpA, *N* (%)	4 (2.7)	0 (0.0)	0.05
Rheumatoid arthritis, *N* (%)	5 (3.4)	1 (0.6)	0.11
Psoriatic arthritis, *N* (%)	2 (1.4)	1 (0.6)	0.61
Fibromyalgia, *N* (%)	3 (2.1)	6 (3.8)	0.50
Juvenile idiopathic arthritis, *N* (%)	2 (1.4)	1 (0.6)	0.61
Gout, *N* (%)	4 (2.7)	1 (0.6)	0.20
Reactive arthritis, *N* (%)	3 (2.0)	0 (0)	0.11
Other arthritis, *N* (%)	3 (2.1)	3 (1.9)	1.00

*Anti-CD74 IgA* anti-CD74 immunoglobulin A, *axSpA* axial spondyloarthritis, *BMI* body mass index, *CRP* C-reactive protein, *MC* Modic changes

The median anti-CD74 IgA was 1.5 U/ml (IQR 0.7; 3.0) in the participants with MC and 1.6 U/ml (IQR 0.6; 2.9) in the participants without MC. Negative and positive controls from the ELISA-based kit confirmed the correct employment of the test (supplemental Table S1). Only four participants had elevated anti-CD74 IgA, all of them belonging to the group without MC. All four of these participants had normal MRI, without any suspicious imaging findings of axSpA. There were four participants with self-reported axSpA who did not have elevated anti-CD74 IgA. One of the self-reported axSpA participants was confirmed with MRI, whereas two had low suspicion of axSpA and one had normal MRI findings. The exact anti-CD74 IgA values for each patient with their MRI manifestation can be found in the supplement (Table S2).

**Fig. 1:**
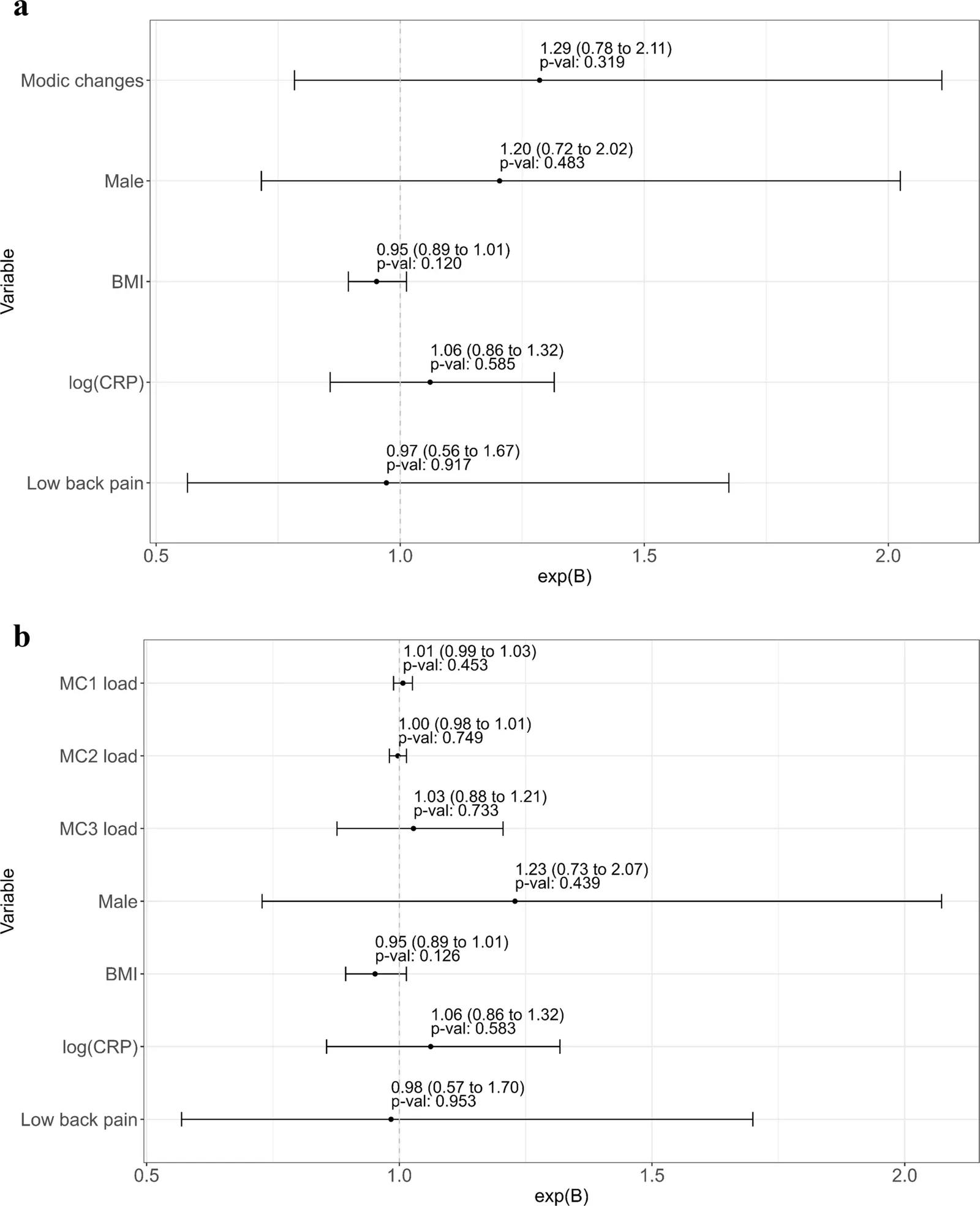
**a** Association of anti-CD74 IgA with Modic changes in linear regression model adjusted for potential confounders (sex, BMI, CRP, presence of low back pain). The four self-reported axSpA patients are excluded from these models. **b** Association of anti-CD74 IgA with Modic change load in linear regression model adjusted for potential confounders (sex, BMI, CRP, low back pain). The four self-reported axSpA patients are excluded from these models. BMI = body mass index, CRP = C-reactive protein; exp (B) = exponential regression coefficient; MC = Modic changes

There was no statistically significant association between MC and continuous anti-CD74 IgA after adjustment for confounders (Exp(B)) = 1.3, 95% CI 0.8 to 2.1) (Fig. 1a). The exponentiated regression coefficient (Exp(B)) corresponds to the expected multiplicative change in the median of anti-CD74 IgA. This implies that in our analysis, the median of anti-CD74 IgA would numerically increase by 1.3 when the diagnosis changes from MRI without MC to MRI with MC. An additional model with differentiation for MC type and MC load (Fig. 1b) did not find a statistically significant association either.

## Discussion

We found no association between participants with MC and anti-CD74 IgA. Anti-CD74 IgA was not elevated to levels seen in axSpA in any of the included participants with MC. Even in MC1 lesions, which most closely resemble axSpA lesions, there was no association.

The major limitation of our study is that we did not compare serum anti-CD74 IgA levels of patients with MC to patients with axSpA. A further important limitation is the long freezing period which could affect the anti-CD74 IgA stability. However, in our previous SCQM cohort study where we used the same ELISA kit for anti-CD74 IgA measurement, serum storage was up to 7 years, without notable lower frequency of positive anti-CD74 IgA results compared to other cohorts [9, 11, 18].

As our study population was initially selected to study MC, we had only four cases of self-reported axSpA in our population and Human Leucocyte Antigen-B27 (HLA-B27) status is unknown. Furthermore, these cases were most probably the result of either wrong diagnosis in the past or misunderstanding of the questionnaire (two participants self-reportedly had every autoimmune arthritis that was asked for), only one of these participants had MRI findings that were highly suggestive of axSpA. None of these participants had elevated serum anti-CD74 IgA levels like those reported in axSpA. However, to avoid confounding through these patients, we excluded them from the association analysis. A direct comparison to axSpA cohorts, like the Swiss Clinical Quality Management (SCQM) cohort [18], is, due to large differences in timing and type of data collection, not useful. Further, we have no information about the thoracic and cervical spine. We therefore do not know if participants without MC or participants with MC had (additional) MCs in the upper part of the spine, although these are rare [21].

Nevertheless, as to date, there is no study directly comparing serum anti-CD74 IgA in MC vs axSpA; our study gives valuable information. If other studies had patients with MC in their control groups, it has not been reported [6–12, 14]. This is the first study to show that anti-CD74 IgA levels are not elevated in patients with MC compared to healthy controls. As macrophage migration inhibitory factor (MIF), the ligand for CD74, has been reported to be a potential biomarker in MC [22, 23], with conflicting results, it was important to show that anti-CD74 IgA does not distinguish MC and controls. In axSpA, MIF has been shown to drive osteoblastic mineralization [24], whereas a rationale for increased [23] or decreased MIF [22] in MC is not known.

Of note, many patients with axSpA might develop degenerative spinal changes over time. The additional degenerative changes can be a reason for pain despite an effective therapy for axSpA [25]. In those patients, anti-CD74 IgA may not be helpful.

In conclusion, the usefulness of anti-CD74 IgA as a diagnostic tool needs to be evaluated in a comparative study between MC and axSpA. If its diagnostic value would be confirmed, anti-CD74 IgA could help to differentiate patients with MC from patients with axSpA at the time point of diagnosis in patients with ambiguous MRI.

## Supplementary Information

Below is the link to the electronic supplementary material.

ESM 1 (XLSX 27.3 KB )

## Data Availability

NFBC data are available from the University of Oulu, Infrastructure for Population Studies. Permission to use the data can be applied for research purposes via an electronic material request portal. In the use of data, we follow the EU general data protection regulation (679/2016) and the Finnish Data Protection Act. The use of personal data is based on a cohort participant’s written informed consent in their latest follow-up study, which may cause limitations to its use. Please, contact the NFBC project center (NFBCprojectcenter@oulu.fi) and visit the cohort website (www.oulu.fi/nfbc) for more information.
